# The Vulnerability of the Apices of the Lungs

**Published:** 1901-06-22

**Authors:** 


### The Vulnerability of the Apices of the Lungs.

Many theories have been put forward to explain the tendency of tuberculous disease to affect the apices in preference to other portions of the lungs, none of which, it must be confessed, are entirely convincing. From a careful review of the whole subject Dr. Colbeck and Dr. Eric Pritchard[1] come to the conclusion that the cause of this predisposition is to be sought in the mechanical conditions under which respiration is carried on in those portions of the lungs, conditions the effects of which are greatly aggravated in certain forms of chest, those very forms indeed which have come from clinical experience to be spoken of as examples of the "phthisical chest."

The first thing which they insist upon is that the dome of lung which projects above the clavicles and upper ribs is practically outside the bony thorax, and that unless the thoracic cavity in this position be closed in by strong and well-developed muscles, the respiratory movements of this portion of the lung tend to be inverted.

It sometimes happens that in the course of operations in the supraclavicular fossa the upper surface of the pleural sac is exposed, so that the movements of the subjacent lung can be seen, and it has been noticed in such cases that the range of movement of this part of the lung is very considerable. But in these operations, and in all cases in which this portion of the lung is not well supported by the muscles by which it is normally surrounded, the movements of the apex must, say the authors of this paper, be inverted as regards their relation to the normal respiratory rhythm, inasmuch as the apex must be sucked into the thorax during each inspiration and protruded during expiration. Under normal conditions the support afforded to the apices of the lungs by the soft parts and the sternal ends of the clavicles when the supraclavicular muscles contract is probably sufficient to permit of the inflation of the apices during inspiration. But if the support afforded by the soft parts be diminished, the inspiratory inflation of the apices becomes correspondingly lessened, and, as this loss of support increases, a time must come when the movements of the apices becomes inverted.

The authors suggest, then, that the explanation of the tendency of tuberculosis to become localised at a point in the upper lobe, about 1 inch to $1\frac{1}{2}$ inch below the true apex, a site that is determined by the extent of the projection of the apex above the border of the first rib, is that here there is a neutral spot, a point of quiescence, between the two currents. Here, they say, is the neutral territory between the opposing currents formed on the one hand by the inverted movements of the apex and on the other by the normal movements of the rest of the lung. In other words, if foreign matter, including bacilli, can collect anywhere in the lung, it is at this site that the collection is most likely to take place, and as it is reasonable to believe that any portion of the lung which, as is the case here, is not functionally active would be likely also to be not so highly resistent as the rest to morbific influences, we have in these circumstances a good explanation of the tendency of tuberculosis to attack this particular spot in preference to the rest of the lung. This conclusion, be it observed, is not of merely academic interest. The relation of a particular form of chest to an especial tendency to inverted movement of the apices of the lungs, coupled with the undoubted relation of this particular form of the chest to a tendency to tuberculosis, bring us at once to a very practical application of the theory.

That the bacillus of tuberculosis is in itself comparatively harmless except in the presence of some predisposing condition in the patient is now well recognised, and one of the mysteries of the whole subject hinges around the question, what are the predisposing causes which enable this organism to take root, and especially what is the local peculiarity in this bit of lung which makes it so often the landing stage through which the whole organism becomes invaded? If, however, the authors of this paper are correct in their conclusions, and if the key to the mystery is in many cases to be found in a lack of proper development in the muscular and bony frame by which the apices of the lung ought to be supported, the practical application of their teaching is very obvious, for the proper development of the chest and its muscles must then be regarded as capable of removing one great predisposing cause of tuberculosis. With this view fully impressed upon us we cannot fail to be convinced of the importance of all such muscular training as shall lead to the proper development of the muscles of the shoulder girdle. To this end the authors suggest that tree-climbing, ball-throwing, horizontal ladder and bar exercises—indeed, any muscular manœuvre which brings into play the great pectoral muscles—should be sedulously and systematically carried out with the object of rectifying that particular peculiarity of the chest—deformity we might well call it—which universal experience has shown to be so often associated with consumptive tendencies.

[1] Lancet, June 8.

